# Differential Regulation of CsrC and CsrB by CRP-cAMP in *Salmonella enterica*

**DOI:** 10.3389/fmicb.2020.570536

**Published:** 2020-10-14

**Authors:** Youssef El Mouali, Guillem Esteva-Martínez, David García-Pedemonte, Carlos Balsalobre

**Affiliations:** Department of Genetics, Microbiology and Statistics, School of Biology, Universitat de Barcelona, Barcelona, Spain

**Keywords:** CsrC, post-transcriptional regulation, sRNA, CRP-cAMP, growth phase regulation, Spot 42

## Abstract

Post-transcriptional regulation mediated by regulatory small RNAs (sRNAs) has risen as a key player in fine-tuning gene expression in response to environmental stimuli. Here, we show that, in *Salmonella enterica*, the central metabolic regulator CRP-cAMP differentially regulates the sRNAs CsrB and CsrC in a growth phase-dependent manner. While CsrB expression remains unchanged during growth, CsrC displays a growth phase-dependent expression profile, being weakly expressed at the logarithmic growth phase and induced upon entry into stationary phase. We show that CRP-cAMP contributes to the expression pattern of CsrC by repressing its expression during the logarithmic growth phase. The CRP-cAMP mediated repression of CsrC is independent of SirA, a known transcriptional CsrB/CsrC activator. We further show that the sRNA Spot 42, which is derepressed in a Δ*crp* strain, upregulates CsrC during logarithmic growth. We propose a model where the growth-dependent regulation of CsrC is sustained by the CRP-cAMP-mediated repression of Spot 42. Together, our data point toward a differential regulation of the sRNAs CsrB and CsrC in response to environmental stimuli, leading to fine-tuning of gene expression via the sequestration of the RNA-binding protein CsrA.

## Introduction

Bacteria need to adapt rapidly to changing environmental conditions, which is particularly crucial for pathogenic bacteria during the process of infection. While transcription plays a major role in the regulation of gene expression, bacteria display a plethora of post-transcriptional mechanisms that allow the fine-tuning of gene expression in response to environmental cues. *Salmonella enterica* has been investigated extensively with respect to gene regulation and has become a model for the study of post-transcriptional RNA-mediated regulation.

Among the described post-transcriptional regulation mechanisms, a prominent role has been attributed to sRNAs that modulate gene expression primarily via binding to target mRNAs (Hör et al., 2020). However, sRNAs can also regulate gene expression at the post-translational level, such as the two sRNAs CsrB and CsrC. These sRNAs contain several stem loop structures with GGA motifs in their loop regions, enabling them to bind CsrA. CsrA is a widely conserved RNA-binding protein that inhibits translation by binding to GGA motifs around the ribosome-binding site of target mRNAs (Romeo and Babitzke, 2018). Binding of CsrB and CsrC to CsrA titrates CsrA away from its target mRNAs, thereby counteracting its inhibitory activity (Liu et al., 1997; Weilbacher et al., 2003).

CRP is a transcription factor that acts as a metabolic sensor and becomes active upon binding to the intracellular second messenger cAMP (cyclic adenosine monophosphate) (Görke and Stülke, 2008). Interaction of CRP-cAMP with DNA leads to activation or repression of its target genes. In *E. coli*, it has been shown that CRP-cAMP represses the expression of both CsrB and CsrC (Pannuri et al., 2016). While repression of CsrB occurs through an indirect mechanism, the repression of CsrC occurs by direct binding of CRP-cAMP to the promoter region of *csrC*, where it competes with the *csrC* activator UvrY (Pannuri et al., 2016). In *Salmonella*, the homolog of the two-component system BarA-UvrY is BarA-SirA (Johnston et al., 1996), which, as in *E. coli*, it also positively regulates the expression of CsrB and CsrC (Teplitski et al., 2003; Fortune et al., 2006; Martínez et al., 2011). Expression studies using *S. enterica* cultures on solid media indicates that CRP-cAMP, in contrast to its role described in *E. coli*, positively regulate the expression of CsrB and CsrC via upregulation of *sirA* (Teplitski et al., 2006). In *S. enterica* other sRNAs, such as CyaR and Spot 42, are also regulated by CRP-cAMP, acting as an activator for CyaR and as a repressor for Spot 42 (Papenfort et al., 2008; El Mouali et al., 2018).

In this study we investigated the role of CRP-cAMP in the expression of the sRNAs CsrB and CsrC in the model organism *S. enterica* using liquid cultures. We describe that CsrC, but not CsrB, display a growth-dependent expression pattern. CsrC expression is silenced during logarithmic growth and highly expressed upon entry into stationary phase, while CsrB expression seems to be constitutive through the growth curve. CRP-cAMP plays a relevant role in this growth-dependent regulatory network. CRP-cAMP differentially regulates the levels of CsrB and CsrC as it does not affect CsrB expression but represses CsrC expression during logarithmic growth. Remarkably, while SirA is required for full expression of CsrC, the CRP-cAMP-mediated repression during logarithmic growth seems to be independent of SirA-mediated regulation. Our data further indicates that Spot 42 contributes to the CRP-cAMP-induced repression of CsrC, suggesting that CRP-cAMP and Spot 42 converge into the differential regulation of CsrB and CsrC in *Salmonella*.

## Results

### CRP-cAMP Represses CsrC Levels at the Logarithmic Growth Phase

**FIGURE 1:**
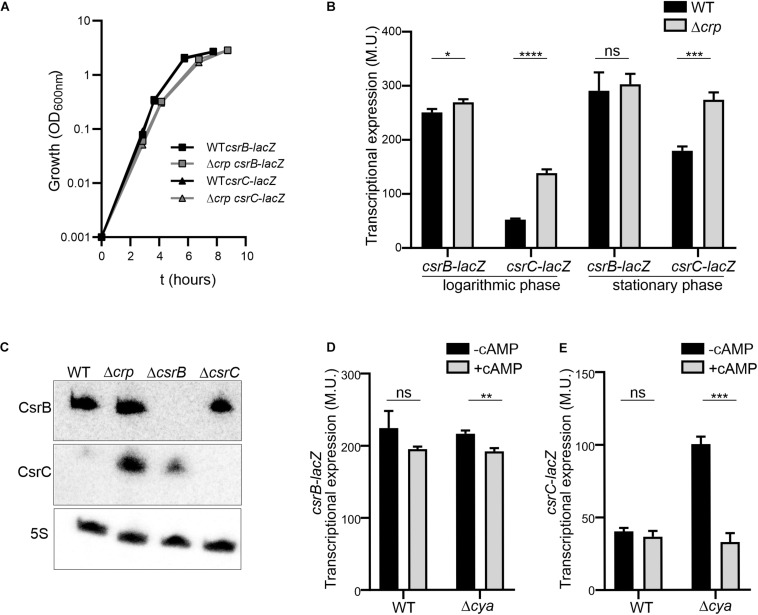
The expression of *csrC* is repressed at the logarithmic growth phase in a CRP-cAMP mediated manner. **(A)** Growth curve in LB at 37°C of *Salmonella* SV5015 strain (WT) and its isogenic Δ*crp* mutant, carrying either a plasmid fusion of *csrB-lacZ* or *csrC-lacZ*. **(B)** Transcriptional expression of *csrB* and *csrC*. Samples from cultures in A were taken at either an OD_60__0_ _nm_ of 0.4 or an OD_60__0_ _nm_ of 2.0 for determination of the β-galactosidase activity in Miller units (M.U.). **(C)** Northern blot analyses of the *csrB* and *csrC* transcripts. Total RNA samples from cultures of WT (SV5015) and its derivatives Δ*crp*, Δ*csrB*, and Δ*csrC* grown in LB up to an OD_60__0_ _nm_ of 0.4 were analyzed. Detection of the 5S transcript was used as a control. Transcriptional expression of *csrB* **(D)** and *csrC* **(E)** in the *cya* mutant strain and chemical complementation. Cultures of WT, Δ*crp* and Δ*cya* derivatives were grown in LB either in the absence or in the presence of cAMP (5 mM) at 37°C up to logarithmic phase (OD_60__0_ _nm_ of 0.4) and samples were taken for β-galactosidase measurement. In B, C and D; β-galactosidase activity was determined for three independent cultures, average and standard deviation are presented. *p < 0.05; **p < 0.01; ***p < 0.001; ****p < 0.0001; ns, not significant.

In order to study the CRP-cAMP mediated regulation of CsrB and CsrC in *Salmonella*, the expression of both sRNAs was monitored using transcriptional reporter fusions. The regulatory elements controlling CsrB and CsrC expression in *Salmonella* have been identified *in silico* and characterized experimentally (Martínez et al., 2014). Accordingly, the described regulatory regions of CsrB (−403, +18) and CsrC (−347, +60) were cloned as *lacZ* transcriptional fusions in the pQF50 vector (Farinha and Kropinski, 1989), allowing to monitor the levels of CsrB and CsrC during growth. The contribution of CRP-cAMP was assessed by determining the expression level of CsrB and CsrC in wild-type (WT) and in a Δ*crp* mutant strain, lacking the transcriptional factor CRP. In rich media, no differences in growth rate could be observed in WT and Δ*crp* strains carrying either *csrB*-*lacZ* or *csrC*-*lacZ* (Figure 1A). Transcriptional expression was monitored during logarithmic growth (OD_60__0_ _n__m_ 0.4) and upon entry into stationary phase (OD_60__0_ _n__m_ 2.0). In the WT, CsrB expression was apparently identical in the two growth phases (Figure 1B). By contrast, CsrC displayed a growth-dependent expression pattern, being less expressed during logarithmic growth and being induced after entering stationary phase (Figure 1B). Remarkably, CRP-cAMP contributes to the CsrC growth-dependent expression pattern. In Δ*crp*, the expression of CsrC is induced when compared to WT at the logarithmic phase and to a lesser extent at the stationary phase, indicating that CRP-cAMP represses CsrC expression (Figure 1B). By contrast, no effect on CsrB regulation by CRP-cAMP was observed. These results were unexpected since it was previously described that CRP-cAMP acts as an activator of both CsrB and CsrC expression when *Salmonella* is grown on LB agar media (Teplitski et al., 2006). To discern if the discrepancy could be consequence of differences in the genetic constructs used to monitor gene expression, a similar experiment as in Teplitski et al. (2006) was performed using our strains. The transcriptional expression was monitored after growth on LB agar media. Consistently with the previous report, the transcriptional expression of CsrB and CsrC was strongly diminished in a Δ*crp* derivative strain compared to WT when *Salmonella* cells were grown on LB agar media as noted by the white colony phenotype of Δ*crp* when compared to the blue colony phenotype of WT (Supplementary Image S1; Teplitski et al., 2006). Altogether, CRP-cAMP seem to be required for activation of the expression of CsrB and CsrC in solid media, while it acts as a repressor of CsrC particularly at logarithmic growth phase. This indicates that CRP-cAMP-mediated regulation of CsrB and CsrC is dependent on the growth conditions.

The differential regulation of CsrC and CsrB by CRP-cAMP at the logarithmic growth phase was further corroborated by the direct RNA detection of CsrB and CsrC. In the WT, CsrC transcript was barely detected, indicating that CsrC expression is tightly silenced. In the Δ*crp* strain, high levels of CsrC were detected indicating that CRP-cAMP is involved in the CsrC silencing during logarithmic growth (Figure 1C). Contrasting the CRP-cAMP-dependent changes in CsrC levels, CsrB was not influenced by knockout of Δ*crp* (Figure 1C), thereby agreeing with the results of our transcriptional fusions (Figure 1B). In addition, deletion of Δ*csrB* cause a mild upregulation of CsrC, presumably by affecting the positive feed forward loop that free CsrA protein exerts on its repressors CsrB and CsrC (Romeo and Babitzke, 2018).

CRP becomes active upon binding to cAMP, which is produced by the adenylate cyclase Cya. Therefore, absence of *crp* or *cya* should display similar expression profiles. Accordingly, the expression of CsrC in logarithmic growth phase is induced in the Δ*cya* derivative strain when compared to WT while no change was observed in the expression of CsrB (Figure 1D). To further confirm the involvement of CRP-cAMP in CsrC regulation, chemical complementation of Δ*cya* was carried out by ectopic addition of cAMP. The addition of cAMP repressed the expression of CsrC in the Δ*cya* derivative strain while no effect was observed in CsrB expression (Figure 1E). Our data indicate that CRP-cAMP is involved in the growth-dependent regulation of CsrC by repressing its expression.

### CRP-cAMP-Mediated Repression of CsrC via a SirA-Independent Pathway

**FIGURE 2:**
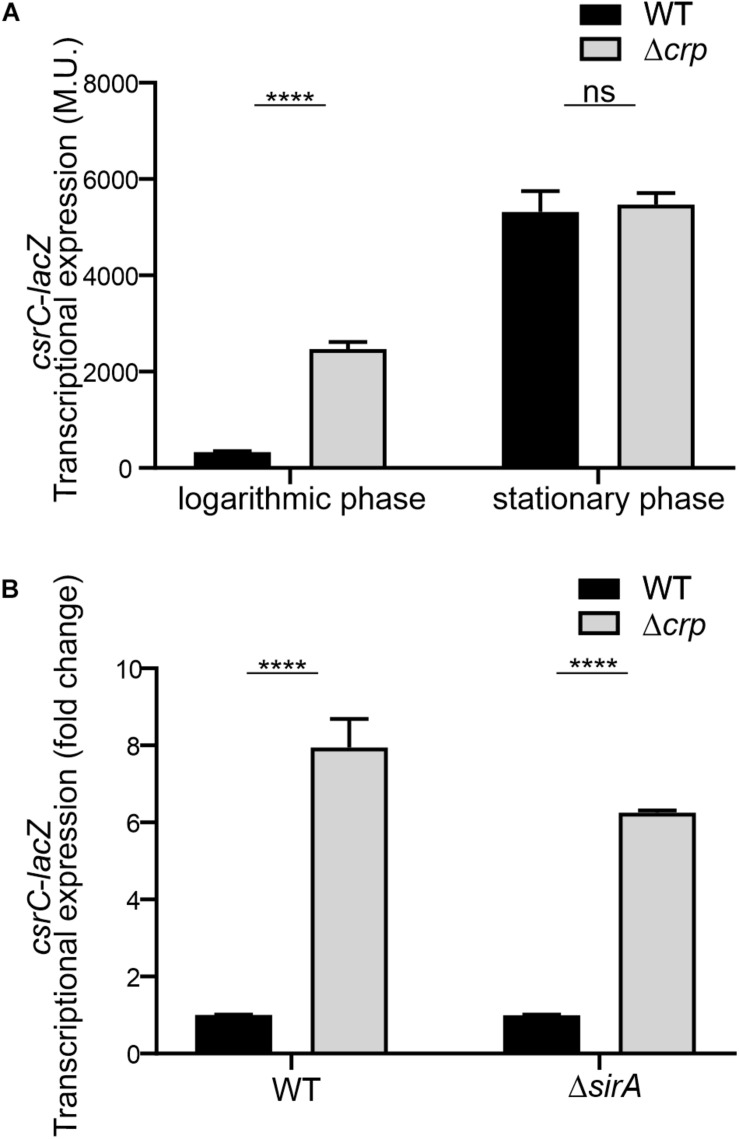
CRP-cAMP mediated repression of *csrC* is independent of SirA. **(A)** Transcriptional expression of chromosomal *csrC*-*lacZ* fusion was monitored in WT and Δ*crp* mutant genetic backgrounds. Cultures were grown in LB at 37°C up to an OD_60__0_ _nm_ of either 0.4 or 2.0. **(B)** Transcriptional expression of the chromosomal *csrC-lacZ* fusion was monitored in WT, Δ*crp*, Δ*sirA*, and Δ*crp*Δ*sirA* genetic backgrounds. The transcriptional expression is shown in relative values. In each case, the reference (*crp*^+^, WT) was set as 1.0. Miller units of the *crp*^+^ backgrounds were 308 ± 19 and 28 ± 0.8 for WT and Δ*sirA*, respectively. β-galactosidase activity was determined for three independent cultures, average and standard deviation is presented. ****p < 0.0001; ns, not significant.

To further characterize the CRP-cAMP mediated regulation of CsrC, a chromosomal *csrC*-*lacZ* fusion was generated. In agreement with earlier results, chromosomal *csrC*-*lacZ* has a growth dependent expression pattern, where it is lowly expressed at the logarithmic growth phase and induced upon entry into early stationary phase (Figure 2A). Interestingly, we further observed an eightfold induction of the chromosomal *csrC-lacZ* fusion in the Δ*crp* background when compared to the WT, which was only true during logarithmic growth (Figure 2A).

The BarA-SirA two-component system was described to positively regulate the expression of CsrC (Teplitski et al., 2006). The possible involvement of BarA-SirA in the regulation of CsrC by CRP-cAMP was assessed. SirA deletion leads to a decrease of the overall transcriptional expression of the chromosomal *csrC*-*lacZ* fusion, 28.2 ± 0.8 Miller units in Δ*sirA* compared to 308.8 ± 19.3 Miller units in WT. Interestingly, despite the overall lower expression levels of CsrC in the Δ*sirA* background, the deletion of Δ*crp* led to a sixfold increase in the transcriptional expression of *csrC*-*lacZ* compared to WT (Figure 2B), indicating that CRP-cAMP modulates *csrC* expression via a SirA-independent mechanism.

**FIGURE 3:**
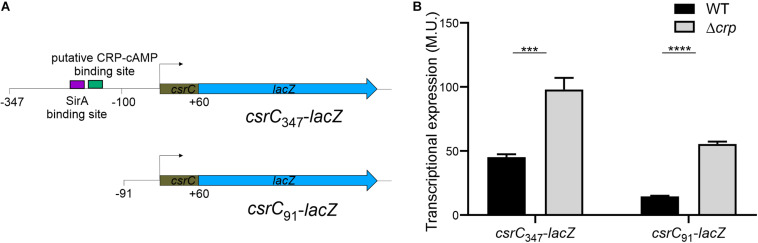
CRP-cAMP represses CsrC in absence of SirA binding site. **(A)** Diagram of the *lacZ* transcriptional fusions present in the pQF*csrC* and pQF*csrC-91* plasmids. The *csrC* upstream sequences cloned in pQF50, –347/ + 60 and –91/+60, are depicted. The relative position of the SirA binding site and the putative CRP-cAMP binding site are indicated with a purple and green rectangle. **(B)** Transcriptional expression of the *lacZ* fusions described in A in WT and Δ*crp* genetic backgrounds. In all cases, cultures were grown in LB at 37°C up to an OD_60__0_ _nm_ of 0.4. β-galactosidase activity was determined for three independent cultures, average and standard deviation are presented. ***p < 0.001; ****p < 0.0001.

In *E. coli*, it was proposed that CRP-cAMP represses CsrC expression through binding to the upstream region of *csrC* and competing with UvrY, the SirA homolog (Pannuri et al., 2016). In *Salmonella*, SirA induces CsrC expression by binding ∼160–168 bp upstream of the *csrC* promoter (Figure 3A; Martínez et al., 2014). Alignment of the upstream promoter regions of CsrC from *E. coli* and *Salmonella* showed that SirA binding site is conserved, whereas the CRP-cAMP binding site displays a lesser extent of conservation (Supplementary Image S2). To characterize if CRP-cAMP binding to the *csrC* promoter is required for the described regulation, an additional *csrC*-*lacZ* fusion was generated where the putative binding sites of these transcriptional factors are not present. As shown in Figure 3A, the pQF50 cloned fragment *csrC*_347_-*lacZ* (−347, +60) maintains SirA/CRP binding sites while *csrC*_91_-*lacZ* (−91, +60) does not. Remarkably, in both *csrC*_347_-*lacZ* and *csrC*_91_-*lacZ* an induction of expression is observed in the Δ*crp* strain when compared to WT (Figure 3B). These results suggest that CRP-cAMP represses the expression of CsrC during logarithmic growth phase via a mechanism that is independent of the competition between SirA and CRP for the *csrC* promoter.

### The sRNA Spot 42 Positively Regulates CsrC

**FIGURE 4:**
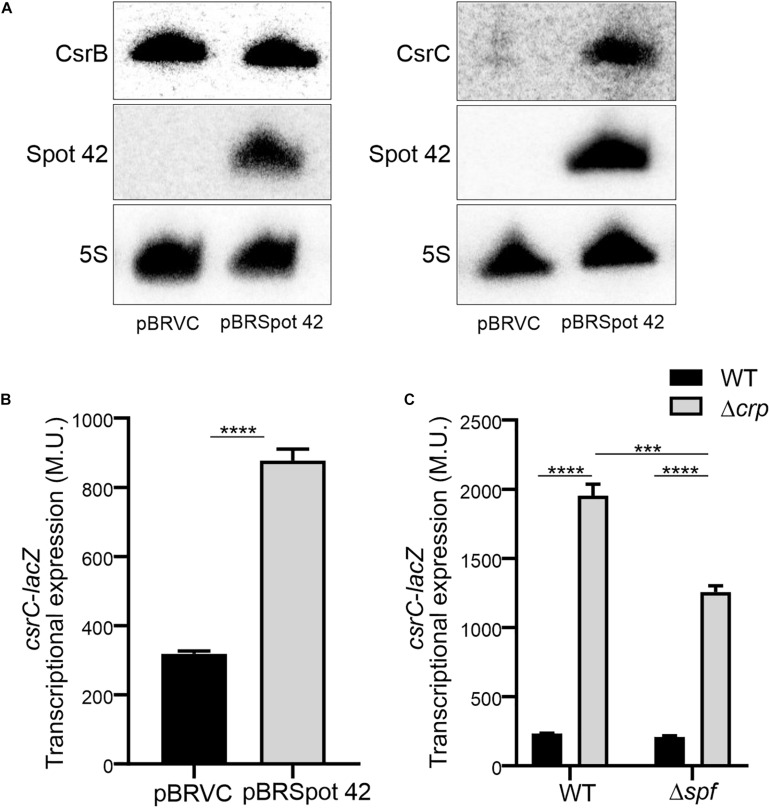
Spot 42 positively regulates the expression of CsrC. **(A)** Northern blot detection of CsrB, CsrC, and Spot 42 was carried out in strains carrying either the pBRplacVC (control) or pBRplac Spot 42 (overexpressing the sRNA Spot 42). 5S RNA was monitored as loading control. **(B)** Transcriptional expression of the chromosomal *csrC*-*lacZ* fusion upon ectopic expression of the sRNA Spot 42 compared to the strain carrying the control vector (pBRplacVC). **(C)** Transcriptional expression of the chromosomal *csrC-lacZ* fusion was monitored in WT, Δ*spf*, Δ*crp*, and Δ*spf*Δ*crp* genetic backgrounds. In all cases cultures were grown in LB at 37°C up to an OD_60__0_ _nm_ of 0.4. β-galactosidase activity was determined for three independent cultures, average and standard deviation is presented. ****p < 0.0001, ***p < 0.001.

In *Salmonella*, CRP-cAMP represses the expression of the regulatory sRNA Spot 42 in a growth-dependent manner (El Mouali et al., 2018). The similarity of the expression profiles of Spot 42 and CsrC, let us to hypothesize that CRP-cAMP might regulate CsrC via modulation of Spot 42 levels. To this end, we determined CsrC expression during logarithmic growth upon ectopic expression of Spot 42. Remarkably, CsrC transcript levels were strongly induced upon Spot 42 overexpression (pBRSpot 42) when compared to the vector control strain (pBRVC) (Figure 4A). In contrast, no accumulation of CsrB was detected upon overexpression of Spot 42 (Figure 4A). Further supporting our hypothesis, Spot 42 seemed to differentially regulate CsrB and CsrC expression (Figure 4A), similar to the described CRP-cAMP-mediated regulation (Figure 1C). To verify this observation, we overexpressed Spot 42 in the chromosomal *csrC*-*lacZ* fusion background, resulting in a threefold induction of CsrC when compared to the vector control strain (Figure 4B). The Spot 42-mediated regulation of CsrC may account for the described repression of CsrC by CRP-cAMP. The contribution of Spot 42 on the induction of CsrC in Δ*crp* was further assessed. The CsrC derepression in a Δ*crp* mutant strain drops in absence of Spot 42 (Δ*spf*) as compared to a Spot 42 proficient strain indicating that Spot 42 contributes to the CsrC derepression in a Δ*crp* background (Figure 4C).

**FIGURE 5:**
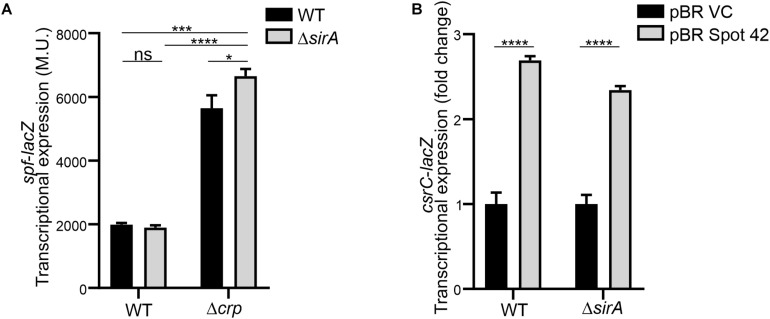
Spot 42 regulates CsrC in a SirA-independent manner. **(A)** Transcriptional expression of the chromosomal *spf*-*lacZ* fusion (Spot 42 expression) was monitored in WT, Δ*crp*, Δ*sirA*, and Δ*crp*Δ*sirA* genetic backgrounds. **(B)** Transcriptional expression of the chromosomal *csrC*-*lacZ* fusion upon ectopic expression of the sRNA Spot 42 compared to the strain carrying the control vector (pBRplacVC) in a WT and a Δ*sirA* backgrounds. The transcriptional expression is shown in relative values. In each case, the reference (pBRVC) was set as 1.0. Miller units of the pBRVC backgrounds were 159 ± 21 and 20 ± 2.2 for WT and Δ*sirA*, respectively. In all cases, cultures were grown in LB at 37°C up to an OD_60__0_ _nm_ of 0.4. β-galactosidase activity was determined for three independent cultures, average and standard deviation are presented. *p < 0.05; ***p < 0.001; ****p < 0.0001; ns, not significant.

Interestingly, it has been described that SirA can bind to the promoter region of *spf* (Zere et al., 2015), suggesting that SirA might be regulating Spot 42. However, transcriptomic data of Δ*sirA* compared to WT in *Salmonella* indicates that Spot 42 it is not regulated by SirA (Colgan et al., 2016). The effect of SirA on Spot 42 expression was assessed, using a chromosomal *spf*-*lacZ* in the presence and absence of SirA in both WT and Δ*crp* genetic backgrounds at logarithmic growth phase. As previously shown, Spot 42 expression is induced in the Δ*crp* mutant compared to WT (El Mouali et al., 2018; Figure 5A). Remarkably, no regulation was observed in the absence of SirA and induction of Spot 42 expression in the Δ*crp* mutant strain is still observed in absence of SirA (Figure 5A). These results indicate that SirA it is not regulating Spot 42 expression under the studied conditions. Furthermore, SirA was not required for the positive effect of Spot 42 on CsrC expression as concluded from *csrC*-*lacZ* expression studies under Spot 42 overexpression in both WT and Δ*sirA*. In both genetic backgrounds, the overexpression of Spot 42 induces the expression of *csrC*-*lacZ* to a similar fold (Figure 5B), consistent with no involvement of SirA in the CRP-cAMP mediated regulation of CsrC.

Altogether, our data indicate that CRP-cAMP differentially regulates CsrB and CsrC at the logarithmic growth phase where it represses specifically CsrC but not CsrB. The CRP-cAMP regulated sRNA Spot 42 arise as new regulator of the Csr regulon in *Salmonella*.

## Discussion

CRP-cAMP is a global regulator initially described to regulate metabolic genes in response to nutrient stimuli. Adding to its more prominent role as transcriptional regulator of mRNAs, CRP-cAMP was also described to regulate the expression of sRNAs such as CyaR, Spot 42, and FnrS (Polayes et al., 1988; Papenfort et al., 2008; De Lay and Gottesman, 2009; Durand and Storz, 2010). CRP-cAMP also modulates the expression of the sRNAs CsrB and CsrC which regulate CsrA protein activity via titration. Previous studies reveal apparent discrepancies in the role of CRP-cAMP on CsrB and CsrC expression. In *E. coli*, CsrB and CsrC are both repressed by CRP-cAMP, being CsrC expression directly repressed by CRP-cAMP via binding competition with the transcriptional activator UvrY (Pannuri et al., 2016). CRP-cAMP activity is modulated, among others, by the phosphorylation state of the EIIA^Glc^, which is involved in glucose transport. When phosphorylated, it stimulates cAMP production, promoting the activity of CRP-cAMP. Surprisingly, dephosphorylated EIIA^Glc^ interacts with CsrD and promotes the degradation of CsrB and CsrC by RNAseE (Leng et al., 2016). In other words, conditions that promote the transcriptional derepression of CsrC and CsrB via CRP-cAMP also promote the degradation of these transcripts (Leng et al., 2016; Pannuri et al., 2016). In contrast, in *Salmonella* CRP-cAMP has been described to play a positive role on CsrB and CsrC expression when it is grown on LB agar media (Teplitski et al., 2006). Although we corroborate these data, we also demonstrate that in LB liquid media, CRP-cAMP differentially regulates CsrB and CsrC. At the logarithmic phase, CsrC but not CsrB is repressed. The alterations in the cell physiology when growing planktonically and within a colony may account for the different regulation described for CsrB and CsrC in *Salmonella.* In addition of being differentially regulated by CRP-cAMP, CsrB, and CsrC depicted distinct expression profiles during the growth curve. CsrB expression seems to be constitutive, whereas, CsrC expression is silenced during logarithmic growth and induced in early stationary phase. The fact that production of CsrC but not CsrB is growth phase dependent indicates that each RNA responds differently to specific environmental inputs. CRP-cAMP is involved in the growth-dependent regulation of CsrC, suggesting that specific physiological signals that alter CRP-cAMP levels would cause alterations in the levels of CsrC and the concomitant alterations of free CsrA levels that would modulate gene expression to promote adaptation to the new conditions. The differential regulation of CsrB and CsrC, not only by CRP-cAMP, but potentially by additional regulators would provide sensitivity to the Csr system. Far from an ON/OFF state, the Csr regulon would display a scale of grays that would allow the bacteria to fine-tune gene expression in response to environmental stimuli. Interestingly, differential regulation of CsrB and CsrC by CRP-cAMP has been reported in *Yersinia pseudotuberculosis*, where CRP-cAMP activates the expression of CsrC and represses the expression of CsrB, highlighting complex species-specific regulation of the Csr system (Heroven et al., 2012).

**FIGURE 6:**
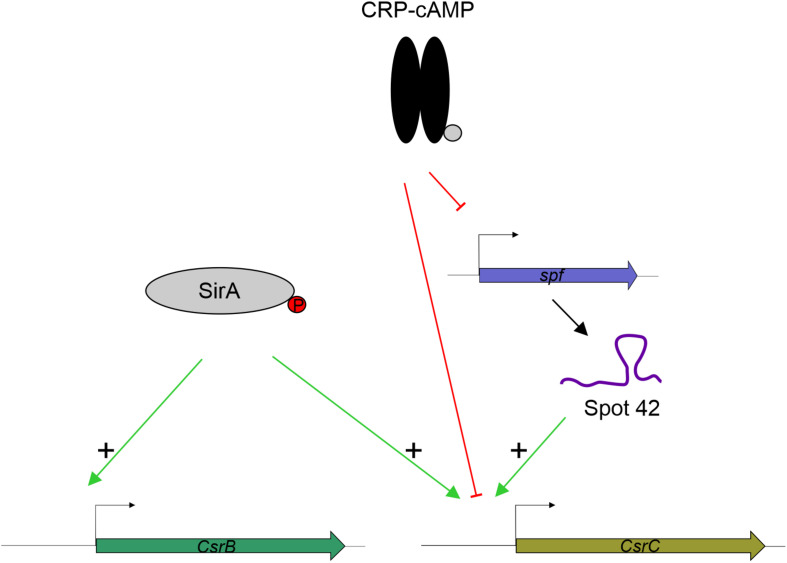
Proposed model for CRP-cAMP mediated repression of CsrC at the logarithmic growth phase. In green, positive regulation is indicated. In red, negative regulation is indicated. SirA, when phosphorylated, it positively regulates CsrB and CsrC. CRP-cAMP represses CsrC and Spot 42. In addition, Spot 42 positively regulates CsrC levels.

SirA is an activator of CsrB and CsrC (Teplitski et al., 2006; Martínez et al., 2011, 2014. While we observe that SirA is required for full activation of CsrC, our data let us conclude that SirA is not involved in the deregulation of CsrC when CRP-cAMP is depleted. The CsrC deregulation was detected both in absence of SirA and when the SirA binding site was removed from the *csrC* promoter. Our data indicate that the sRNA Spot 42, which is transcriptionally repressed by CRP-cAMP at the logarithmic growth phase, positively regulates CsrC expression but it does not affect CsrB (Figure 6). This suggests a model where CRP-cAMP differentially regulates CsrB and CsrC via derepression of the trans-encoded sRNA Spot 42. Of note, Spot 42 does not seem to be solely responsible for the derepression of CsrC in absence of *crp*, as a partial derepression of CsrC is still observed in absence of *crp* and *spf*. Suggesting that, CRP-cAMP additionally represses CsrC expression through a Spot 42 independent mechanism (Figure 6).

The role of Spot 42 in the regulation of CsrC seem to be restricted to logarithmic growth phase and it is not involved in the stationary phase dependent induction. Expression of *csrC*-*lacZ* is induced at stationary phase compared to logarithmic growth phase in both the WT and the Δ*spf* backgrounds (Supplementary Image S3). Consistently, transcriptomic data indicates that the expression level of Spot 42 is downregulated 100-fold upon entry into stationary phase when compared to logarithmic growth (Kröger et al., 2013). In agreement, Spot 42 might be responsible to fine-tune the expression of CsrC in conditions where the CRP-cAMP activity is downregulated similarly as it occurs for the Spot 42-regulated *hilD* mRNA (El Mouali et al., 2018).

In this report, we provide new insights in the link between the CRP-cAMP and Csr regulons in *Salmonella*. We demonstrate that CRP-cAMP differentially regulates CsrB and CsrC. These two sRNAs are considered functionally redundant but our results suggest that they respond distinctly under specific environmental and physiological conditions in *Salmonella*. The presence of CsrB alone would affect the free pool of CsrA to a lesser extent than when both CsrB and CsrC sRNAs are present. This fine-tuning of the free CsrA levels would affect the Csr regulon where high affinity mRNA targets will require reduced amounts of free CsrA whereas low affinity targets will require full derepression of CsrA. We show that CRP-cAMP and its target sRNA Spot 42 contribute to the overall levels of CsrB and CsrC. Its contribution allows *Salmonella* to tightly control the levels of free CsrA in response to environmental stimuli. These features contribute to the versatility of pathogenic bacteria such as the model organism *Salmonella enterica*.

## Materials and Methods

### Bacterial Strains, Plasmids, and Growth Conditions

The bacterial strains, derivatives of *Salmonella enterica* serovar Typhimurium SL1344, were cultivated in Lysogeny broth (LB; tryptone 10 g/l, yeast extract 5 g/l and sodium chloride 10 g/l). When required, media was supplemented with the antibiotics indicated, ampicillin (Amp) 100 μg/ml, chloramphenicol (Cm) 15 μg/ml or kanamycin (Km) 50 μg/ml. Bacterial cultures were inoculated at an OD_60__0_ _nm_ of 0.001, cultures reaching OD_60__0_ _nm_ 0.4 were considered logarithmic growth and reaching OD_60__0_ _nm_ 2.0 was considered early stationary phase. The bacterial strains and plasmids used in this study are listed in Supplementary Tables S1, S2, respectively.

### Genetic Manipulations

Deletion strains were generated by standard gene replacement as previously described (Datsenko and Wanner, 2000). For chromosomal *csrC*-*lacZ* transcriptional fusion. The *csrC*:Cm strain was cured from the antibiotic resistance as previously described (Cherepanov and Wackernagel, 1995). The resulting *csrC*:*frt* strain carrying the FRT scar was further used to obtain chromosomal *csrC*-*lacZ* by integration of pKG136 as previously described (Ellermeier et al., 2002). Chromosomal *spf*-*lacZ* was obtained as for CsrC (El Mouali et al., 2018). Oligonucleotides used for strains construction are listed in Supplementary Table S3.

Plasmidic transcriptional fusions were generated for *csrB* and *csrC*. The regulatory regions of interest were PCR amplified, *Bam*HI/*Hin*dIII digested and ligated within pQF50 (Farinha and Kropinski, 1989). Spot 42 was cloned in pBRplac (Guillier and Gottesman, 2006; Beisel and Storz, 2011). Spot 42 was PCR amplified, *Aat*II/*Eco*RI digested and ligated in pBRplac. Spot 42 was expressed constitutively in *Salmonella* (El Mouali et al., 2018). Oligonucleotides used for cloning are listed in Supplementary Table S3.

### β*-*Galactosidase Activity Assay

Strains of interest were grown to logarithmic growth phase (OD_60__0_ _n__m_ 0.4) or early stationary phase (OD_60__0_ _n__m_ 2.0). β-galactosidase activity was measured as described previously (Miller, 1992). Shortly, 100 μl of culture was added to 900 μl of buffer Z (60 mM Na_2_HPO_4_, 40 mM NaH_2_PO_4_, 10 mM KCl, 1 mM MgSO_4_, and 50 mM 2-mercaptoethanol, pH 7) and cells were lysed by the addition of 10 μl of toluene. Reactions were incubated at 28°C and β-galactosidase activity was measured upon addition of 200 μl of ONPG (4 mg/ml). Reactions were stopped by the addition of 500 μl of Na_2_CO_3_ (1 M). The OD_42__0_ _n__m_ and OD_55__0_ _n__m_ was measured and Miller Units were calculated as previously described (Miller, 1992). β-galactosidase activity determination was performed in technical duplicates for each of three biological replicates.

### Total RNA Isolation and Northern Blot

Strains of interest were grown to logarithmic growth phase (OD_60__0_ _n__m_ 0.4). The biomass of 4 units of OD_60__0_ _n__m_ was collected, and total RNA extracted by classic hot phenol method. Shortly, the cells were resuspended in 600 μl of TE (10 mM Tris-HCl, 1 mM EDTA, pH 8.0) containing 0.5 mg/ml lysozyme. Then, 60 μl of 10% SDS (w/v) was added; mixed by inversion and incubated at 64°C for 1–2 min. After the incubation, 66 μl of sodium acetate pH 5.2 (1 M) was added and mixed by inversion. For RNA extraction, 750 μl of Roti-Aqua phenol was added to the samples, mixed by inversion and incubated at 64°C for 6 min. Upon centrifugation (15 min, 13,000 rpm, 4°C), the top aqueous layer was transferred to a fresh 2 ml microcentrifuge tube, and 750 μl of chloroform was added. The samples were mixed and upon centrifugation (12 min, 13,000 rpm, 15°C), the upper aqueous layer was transferred into a new tube and precipitated with 30:1 mix of ethanol and sodium acetate (1 M, pH 6.5). The samples were incubated for 2–3 h or overnight at −20°C. The samples were then centrifuged and precipitated RNA was resuspended in water and concentration measured by NanoDrop. Samples of 10 μg of total RNA were subjected to electrophoretic separation in Tris-Borate-EDTA (TBE) 8% acrylamide gels containing 8.3 M urea. RNAs were transferred to Hybond N + (GE Healthcare) filters by semi-dry TBE based transference. Transcripts of interest were detected by hybridization with 5′ radiolabeled oligos as probes. Images were obtained with the FLA-5100 imaging system (Fujifilm). Oligonucleotides used as probes are listed in Supplementary Table S3.

### Statistical Analysis

Graph Pad 8.0 software was used for data analysis. Unpaired two-tailed Student’s *t*-test were carried out for two groups comparison and *p* < 0.05 were considered significant.

## Data Availability Statement

The raw data supporting the conclusions of this article will be made available by the authors, without undue reservation.

## Author Contributions

YE, GE-M, and DG-P contributed to the investigation. YE and CB contributed to the conceptualization, investigation, formal analysis and writing the manuscript. All authors contributed to the article and approved the submitted version.

## Conflict of Interest

The authors declare that the research was conducted in the absence of any commercial or financial relationships that could be construed as a potential conflict of interest.
