# Corrigendum: Differential Regulation of CsrC and CsrB by CRP-cAMP in *Salmonella enterica*

**DOI:** 10.3389/fmicb.2021.723431

**Published:** 2021-07-02

**Authors:** Youssef El Mouali, Guillem Esteva-Martínez, David García-Pedemonte, Carlos Balsalobre

**Affiliations:** Department of Genetics, Microbiology and Statistics, School of Biology, Universitat de Barcelona, Barcelona, Spain

**Keywords:** CsrC, post-transcriptional regulation, sRNA, CRP-cAMP, growth phase regulation, Spot 42

In the original article, there was an error in the Funding statement as published. Some of the funders were missing from the original statement.

The corrected Funding statement is shown below.

This work was supported by the Spanish Ministry of Economy and Competitiveness (grant AGL2013-45339R), the Spanish Ministry of Science, Innovation and Universities (MCIU), State Bureau of Investigation (AIE), and European Regional Development Fund (FEDER) (grant PGC2018-096958- B-I00), and the Catalonian Government (grant 2017SGR499). YE was recipient of an APIF fellowship from the University of Barcelona.

The authors apologize for this error and state that this does not change the scientific conclusions of the article in any way. The original article has been updated.

