# Supplementary figures and images for: Differential Regulation of CsrC and CsrB by CRP-cAMP in Salmonella enterica
Source: Front Microbiol. 2020 Oct 14;11:570536. doi: 10.3389/fmicb.2020.570536 (PMC7591399; doi:10.3389/fmicb.2020.570536)

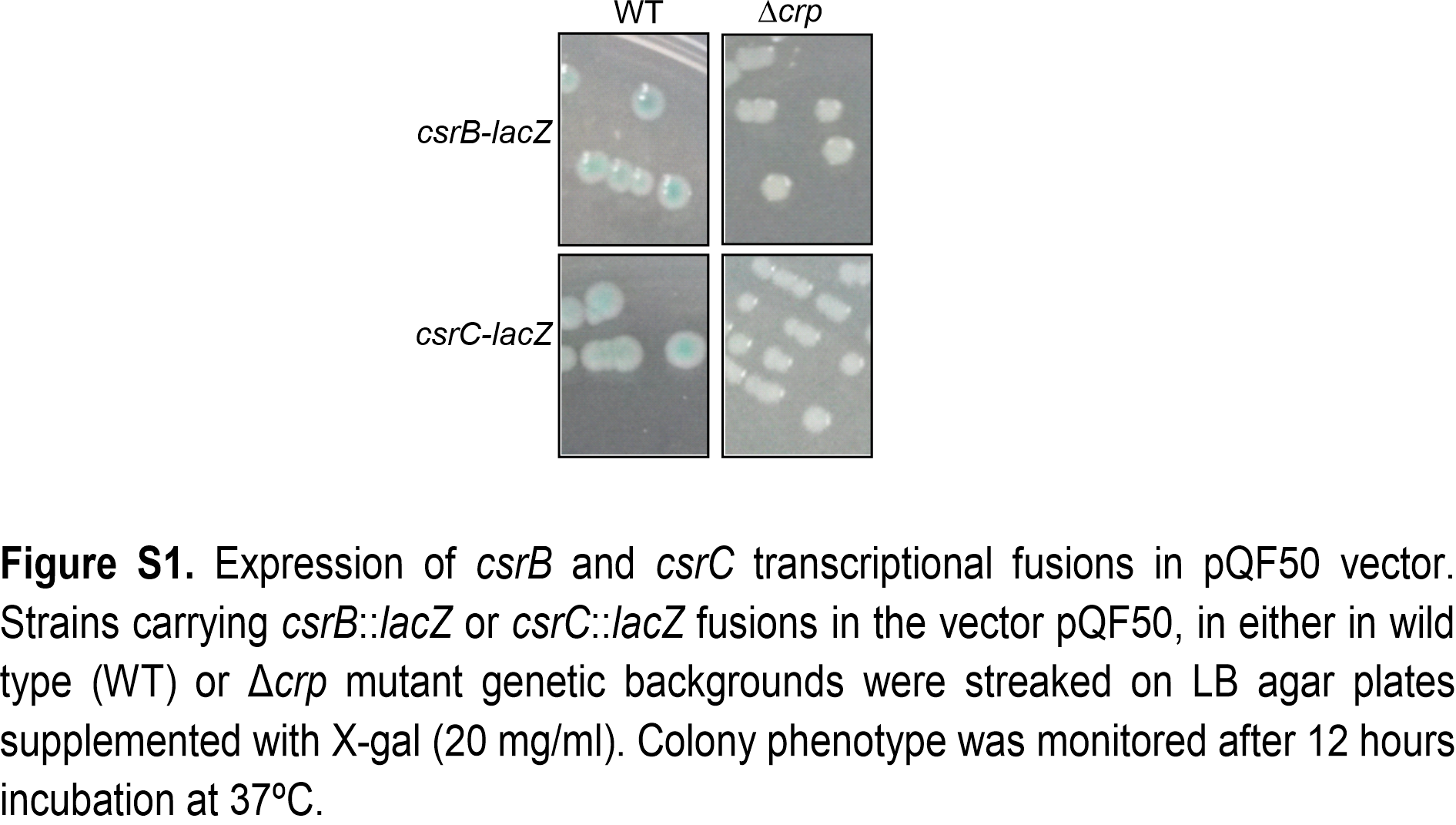

Supplement: Supplementary file 1 [file Image_1.TIF]

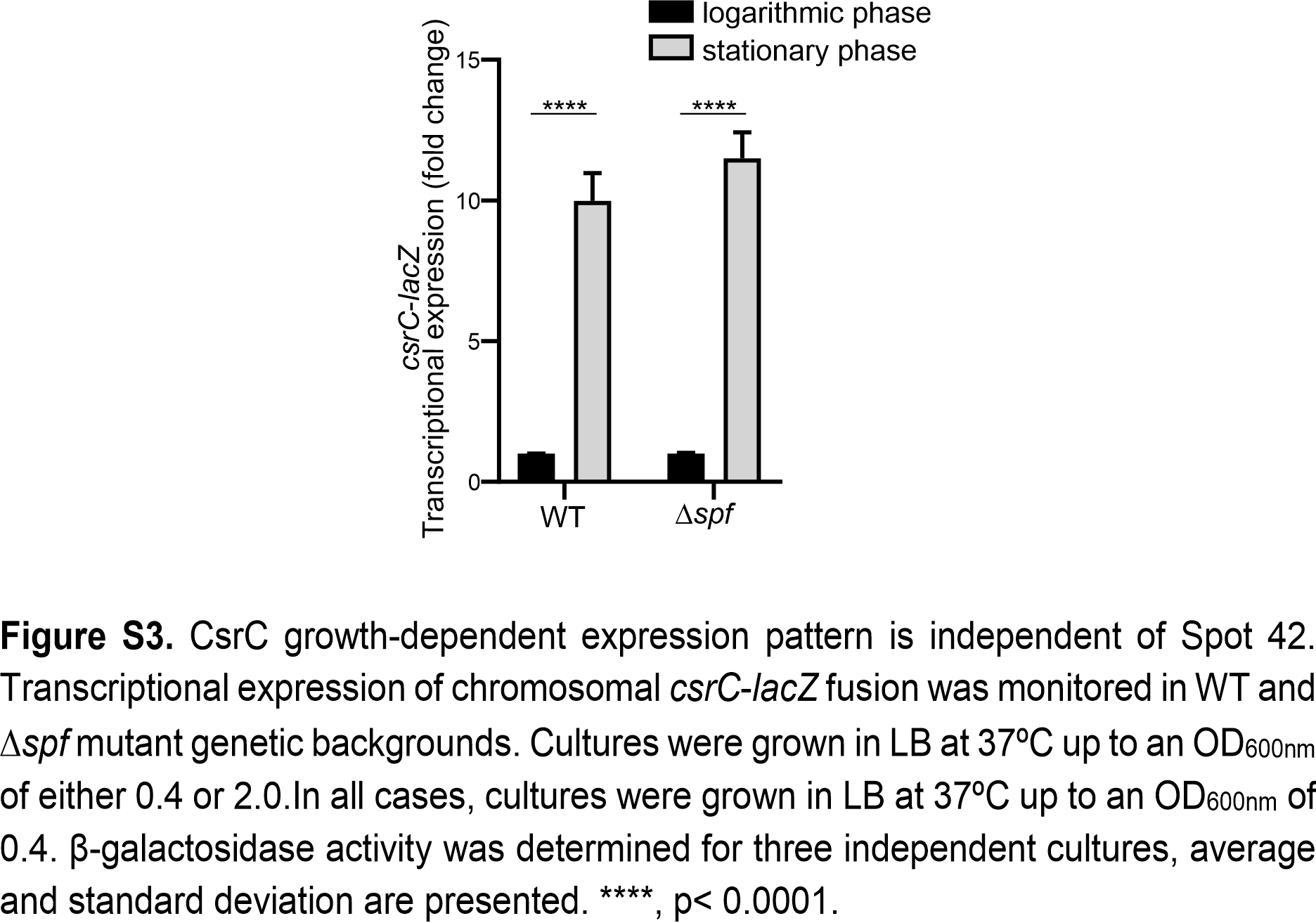

Supplement: Supplementary file 3 [file Image_3.TIF]
