# Supplementary material for: Differential Regulation of CsrC and CsrB by CRP-cAMP in Salmonella enterica
Source: Front Microbiol. 2020 Oct 14;11:570536. doi: 10.3389/fmicb.2020.570536 (PMC7591399; doi:10.3389/fmicb.2020.570536)
Supplement: Supplementary file 4 [file Table_1.pdf]

TABLE S1. Bacterial strains

Strains	Genotype	Source
SV5015	*Salmonella enterica* serovar Typhimurium SL1344 His+	(Baños et al. 2009)
TGC200	SV5015 Δ*crp*:frt	(El Mouali et al. 2018)
TGC201	SV5015 Δ*cya*:frt	(El Mouali et al. 2018)
TGC289	SV5015 pQF50 *csrB-lacZ*	This study
TGC290	SV5015 Δ*crp:frt* pQF50 *csrB-lacZ*	This study
TGC291	SV5015 pQF50 *csrC-lacZ*	This study
TGC292	SV5015 Δ*crp:frt* pQF50 *csrC-lacZ*	This study
TGC293	SV5015 Δ*csrB*::*cm*	This study
TGC294	SV5015 Δ*csrC*::*cm*	This study
TGC295	SV5015 *csrC-lacZ*	This study
TGC296	SV5015 Δ*crp*:*frt csrC-lacZ*	This study
TGC298	SV5015 Δ*sirA*:*cm csrC-lacZ*	This study
TGC299	SV5015 Δ*crp*:*frt* Δ*sirA*:*cm csrC-lacZ*	This study
TGC502	SV5015 pQF50 *csrC91-lacZ*	This study
TGC503	SV5015 Δ*crp:frt* pQF50 *csrC91-lacZ*	This study
TGC300	SV5015 *csrC-lacZ* pBRplac VC	This study
TGC301	SV5015 *csrC-lacZ* pBRplac Spot 42	This study
TGC504	SV5015 Δ*spf*::*cm csrC-lacZ*	This study
TGC505	SV5015 Δ*crp*:*frt* Δ*spf*::*cm csrC-lacZ*	This study
TGC506	SV5015 *csrC-lacZ* Δ*sirA*:*cm* pBRplac VC	This study
TGC507	SV5015 *csrC-lacZ* Δ*sirA*:*cm* pBRplac Spot 42	This study
TGC254	SV5015 *spf-lacZ*	(El Mouali et al. 2018)
TGC255	SV5015 Δ*crp:frt spf-lacZ*	(El Mouali et al. 2018)
TGC508	SV5015 Δ*sirA*:*cm spf-lacZ*	This study
TGC509	SV5015 Δ*crp*:*frt* Δ*sirA*:*cm spf-lacZ*	This study
