# Supplementary material for: Differential Regulation of CsrC and CsrB by CRP-cAMP in Salmonella enterica
Source: Front Microbiol. 2020 Oct 14;11:570536. doi: 10.3389/fmicb.2020.570536 (PMC7591399; doi:10.3389/fmicb.2020.570536)
Supplement: Supplementary file 5 [file Table_2.pdf]

TABLE S2. Plasmids

Plasmids	Antibiotic resistance	Source
pKD3	Amp^R	(Datsenko and Wanner, 2000)
pKD4	Amp^R	(Datsenko and Wanner, 2000)
pKD46	Amp^R	(Datsenko and Wanner, 2000)
pCP20	Amp^R	(Datsenko and Wanner, 2000)
pBR plac	Amp^R	(Guillier and Gottesman, 2006)
pBRplac Spot 42	Amp^R	(El Mouali et al., 2018)
pQF50	Amp^R	(Farinha and Kropinski, 1990)
pQF50 *csrB-lacZ*	Amp^R	This study
pQF50 *csrC-lacZ*	Amp^R	This study
pQF50 *csrC91-lacZ*	Amp^R	This study
pSUB11	Km^R	(Uzzau et al., 2001)
pKG136	Km^R	(Ellermeier et al., 2002)
