# Supplementary material for: Differential Regulation of CsrC and CsrB by CRP-cAMP in Salmonella enterica
Source: Front Microbiol. 2020 Oct 14;11:570536. doi: 10.3389/fmicb.2020.570536 (PMC7591399; doi:10.3389/fmicb.2020.570536)
Supplement: Supplementary file 6 [file Table_3.pdf]

TABLE S3. Oligonucleotides

Primers	Sequence
	**Gene deletions**
*csrB*_P1	GTACAACGAAGCGAACGTCAGGATGATGACGCTTCAGCAGGTGTAGGCTGGAGCTGCTTC
*csrB*_P2	CATCCGTGACAACTTTTCCTGTGACCTTACGGCCTGTTCACATATGAATATCCTCCTTA
*csrC*_P1	GAGGACGCTAACAGGATCAACGACTCAGGATGAGGGTGTAGGCTGGAGCTGCTTC
*csrC*_P2	CTTAACGGGTTCCACCATCCCTGTTGGCTATCAACACGCCCTCATATGAATATCCTCCTTA
*sirA*_P1	CAGGGATACGACGCATTCTTGAAGATATAAAGGGCATTAAAGGTGTAGGCTGGAGCTGCTTC
*sirA*_P2	CCAGGTGAGTCAGCTCAACATCACCATGAATGTTTAATTTACCATATGAATATCCTCCTTA
	**Gene cloning**
*csrB*fw_pQF50	CGCGGATCCTAGGCCACCTGGTCACGC
*csrB*rv_pQF50	CGCAAGCTTCGACTCCCTGTCGACGAAG
*csrC*fw_pQF50	CGCGGATCCGCAATCGGAAGGTAAATGG
*csrCrv*_pQF50	CGCAAGCTTCGCCTCCTGGCGCTCC
*csrCfw91*_pQF50	CGCGGATCCCATGAATTTATAGAAAGG
	**Northern blot probes**
CsrC probe	CATCCTGAGTCGTTGATCCTG
CsrB probe	GGAGGTGTCCTTTAACGCATC
Spot 42 probe	CAAATCCGATTACGTGAAGT
5S_probe	CTACGGCGTTTCACTTCTGAGTTC
